# Supplementary material for: Berries from Luzuriaga radicans Ruiz & Pav.: A Southern Chile Climbing Shrub as a Source of Antioxidants Against Chronic Diseases
Source: Plants (Basel). 2025 Aug 17;14(16):2555. doi: 10.3390/plants14162555 (PMC12389088; doi:10.3390/plants14162555)
Supplement: Supplementary file 1 [file plants-14-02555-s001.zip › plants-3776519-supplementary.pdf]

Supplementary material for the article:

Berries from *Luzuriaga radicans* Ruiz & Pav.: A Southern Chile Climbing Shrub as a Source of Antioxidants Against Chronic Diseases

---

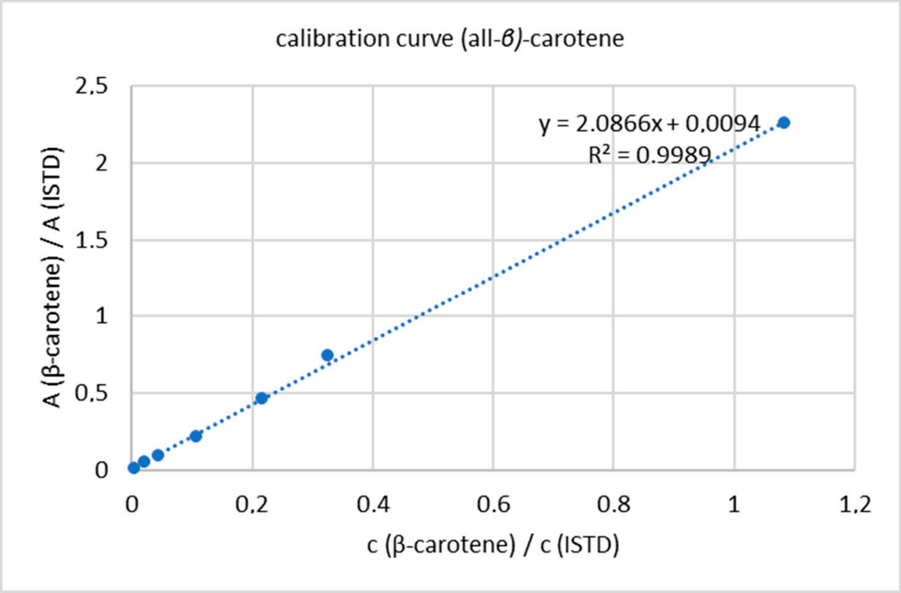

Figure S1. External (all-E)-β-carotene-calibration curve. Quantification was performed by UHPLC-DAD as (all-E)-β-carotene equivalents.

Table S1. Quantified carotenoid contents (mg/kg DW), calculated as (all-E)-β-carotene equivalents.

**Compound**	**Concentration (mg/kg DW)**
(all-*E*)-lutein and (all-*E*)-zeaxanthin	7.95 ± 0.15
Unknown 1	3.23 ± 0.07
ni, xanthophyll isomer MW 568	3.74 ± 0.18
ni, xanthophyll MW 552	3.09 ± 0.18
ni, xanthophyll isomer MW 568	11.84 ± 0.38
ni, xanthophyll MW 600 caprate	4.85 ± 0.15
ni, xanthophyll MW 600 caprate	7.48 ± 0.18
ni, xanthophyll MW 600 laurate	5.39 ± 0.18

ni, xanthophyll MW 568	46.29 ± 2.4
(all-*E*)-*β*-cryptoxanthin	27.65 ± 1.55
ni, xanthophyll MW 552	19.79 ± 1.42
(all-*E*)-violaxanthin laurate	36.00 ± 0.48
ni, xanthophyll MW 552	41.96 ± 1.42
ni, xanthophyll MW 552	25.84 ± 0.74
ni, xanthophyll MW 552	32.56 ± 0.09
ni, xanthophyll MW 552	4.72 ± 0.21
ni, xanthophyll MW 552	3.38 ± 0.04
ni, xanthophyll MW 552	5.05 ± 0.32
ni, xanthophyll MW 552	9.81 ± 0.28
ni, xanthophyll MW 552	35.04 ± 0.33
ni, xanthophyll MW 552	16.71 ± 0.42
ni, xanthophyll MW 552	29.56 ± 2.23
(15-*Z*)-*β*-carotene	117.44 ± 5.37
β-zeacarotene	11.73 ± 1.01
ni, carotene MW 536	11.55 ± 1.62
(13-*Z*)-*β*-carotene	75.94 ± 4.77
ζ-carotene isomer 2	17.44 ± 0.89
(all-*E*)-*β*-carotene	115.07 ± 4.57
ni, xanthophyll MW 552	15.42 ± 1.65
ni, carotene MW 536	6.16 ± 0.42
(9-*Z*)-*β*-carotene	50.87 ± 1.24
γ-carotene	77.69 ± 1.21
(all-*E*)-violaxanthin-laurate myristate / ni, xanthophyll MW 569-dilaurate	3.67 ± 0.25
(*Z*)-Lycopene	40.1 ± 0.54
(all-*E*)-*β*-cryptoxanthin laurate	15.42 ± 0.36
ni, xanthophyll MW 552 laurate	11.2 ± 0.73
(all-*E*)-lycopene	25.95 ± 1.3
(all-*E*)-*β*-cryptoxanthin palmitate	2.88 ± 0.03
Unknown 2	2.93 ± 0.08
**Sum of carotenoids**	**983.4 ± 26.3**

Note: ni: not identified; DW: dry weight.

Figure S2. Negative ESI HPLC-QTOF-MS base peak chromatogram of the hydroethanolic extract from *Luzuriaga radicans*

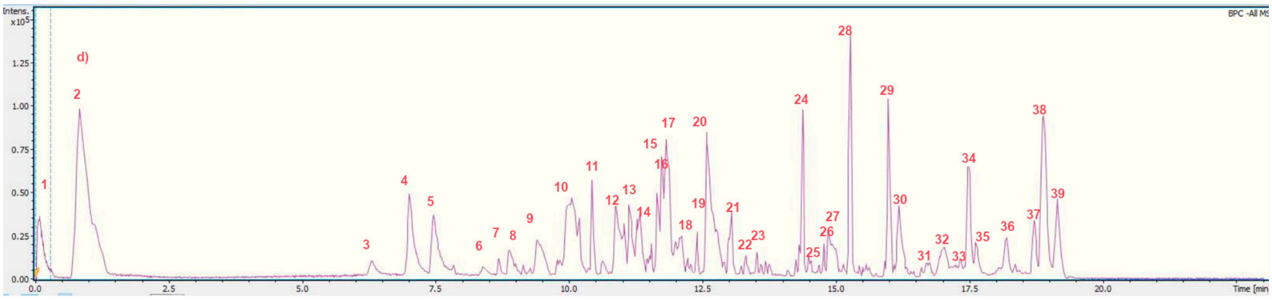

Table S2. Negative ESI HPLC-QTOF-MS identification of the hydroethanolic extract from *Luzuriaga radicans*

Peak #	Retention time	Tentative identification	Molecular formula [M-H]	Measured mass (m/z)	Theoretical mass (m/z)	Accuracy (ppm)	Ions MS^n	type
1	1.38	Na formiate (internal standard)	$NaC_2H_2O_4^-$	112.9829	112.9856	3.3	57.3556	
2	1.43	Malic acid	$C_4H_5O_5^-$	133.01355	133.01315	3.008	102.9959	Organic acid
3	6.3	Docosahexaenoic acid	$C_{22}H_{31}O_2^-$	327.2448	327.2330	-36.1	655.4930, 269.0683	Fatty acid
4	7.0	Docosapentaenoic acid	$C_{22}H_{33}O_2^-$	329.2613	329.2486	-38.6	659.5274	Fatty acid
5	7.5	Quinic acid	$C_7H_{11}O_6^-$	191.05563	191.05501	3.2	135.0447	244-293-311
6	8.4	Hydroxycaffeoyl quinic acid	$C_{16}H_{19}O_{10}^-$	371.09866	371.09727	3.7	341.9958, 191.1584, 173.7155	Phenolic acid
7	8.5	Tricin	$C_{17}H_{14}O_7^-$	329.0676	329.0749	3.1	179.7858	Flavonoid
8	9.1	Caffeoyl acid hexoside	$C_{15}H_{17}O_9^-$	341.08786	341.08671	3.3	160.8416, 135.0444	Phenolic acid
9	9.4	3-O-*p*-Coumaroyl quinic acid	$C_{16}H_{17}O_8^-$	337.09283	337.09179	3.0	191.1472, 172.2738, 162.8387, 119.1619	Phenolic acid

9	10.1	p-Coumaroyl acid hexoside	$C_{15}H_{17}O_8^-$	325.09293	325.09179	3.4	163.0390, 162.8383, 119.0491	Phenolic acid
11	10.5	Methyl 10-oxo-8-octadecenoate	$C_{19}H_{33}O_3^-$	309.2321	309.2224	37		Fatty acid
12	11.0	Gallicynoic acid e	$C_{18}H_{29}O_5^-$	325.2110	325.2173	7.1	255.2517, 651.4249	Fatty acid
13	11.2	5-O-*p*-Coumaroyl quinic acid	$C_{16}H_{17}O_8^-$	337.09299	337.09179	3.54	173.6787, 163.3620, 119.6234	Phenolic acid
14	11.4	D-glucitol monolaurate	$C_{18}H_{33}O_5^-$	363.2465	363.2541	13.4	195.1493	Sugar fatty acid
15	11.6	Methyl vanillate glucoside	$C_{15}H_{19}O_9^-$	343.1106	343.1035	-20	709.2114, 328.0851, 259.0788	Fatty acid
16	11.7	5-O-Feruloylquinic acid	$C_{17}H_{19}O_9^-$	367.10376	367.10236	3.81	191.05559	206-249-300
17	11.9	5-O-Caffeoyl quinic acid	$C_{16}H_{17}O_9^-$	353.08783	353.08671	3.17	191.0556, 135.0446, 133.9936	Phenolic acid
18	12.2	Octacosanoic acid, 14,17,18-trihydroxy-20-methyl-, methyl ester	$C_{27}H_{51}O_5^-$	499.4035	499.4368	3.3	255.2512	Fatty acid
19	12.4	Ferulic acid	$C_{10}H_9O_4^-$	193.05020	193.04954	3.45		Phenolic acid
20	12.6	Ascorbyl Stearate	$C_{24}H_{41}O_7^-$	441.2886	441.2858	2.2	372.3068, 233.1707	Sugar fatty acid
21	13.1	Glyceryl Dilaurate	$C_{27}H_{51}O_5^-$	455.3875	455.3895	28.9	112.9817	Sugar fatty acid
22	13.3	Torvoside G	$C_{34}H_{55}O_9^-$	607.3987	607.4004	-22.3	561.3847	Terpene
23	14.1	Tokoronin	$C_{32}H_{51}O_9^-$	579.3313	579.3386	38		terpene
24	14.4	*Unknown*	$C_{34}H_{53}O_{12}^-$	653.3538	653.3543	2.2	583.3652	Terpene
25	14.7	*Unknown*	$C_{33}H_{59}O_6^-$	551.4425	551.4470	8.2	281.2705	Terpene
26	14.9	Kaempferide	$C_{16}H_{11}O_6^-$	299.0568	299.0568	2.53	1075.2509, 375.0811	Flavonoid
27	15.1	Unknown	$C_{15}H_{31}O_6^-$	307.2178	307.2126	-17	508.3581, 220.1000,	sugar
28	15.3	Methyl 9-oxooctadecanoate	$C_{19}H_{25}O_{13}^-$	461.12979	461.12897	1.787		Fatty acid

29	16	9-hydroxy-10,12-octadecadienoic acid	$C_{19}H_{35}O_3^-$	311.2507	311.2436	-26.0	171.1071	Fatty acid
30	16.3	1,2-octadecanediol	$C_{18}H_{37}O_2^-$	285.2713	285.2799	30	112.9822	Fatty acid
31	16.7	Rheidin C	$C_{31}H_{21}O_9^-$	537.1306	537.1191	-17.7	1075.2509, 375.0811	Terpene
32	17	18-hydroxy-oleic acid	$C_{18}H_{32}O_3^-$	295.2281	295.2383	2.5	211.1333	Fatty acid
33	17.2	Unknown	$C_{19}H_{31}O_5^-$	339.2278	339.2177	-29	183.0212	Fatty acid
34	17.5	Caffeic acid	$C_9H_7O_4^-$	179.03442	179.03389	3.008	151.2131	Phenolic acid
35	17.6	Myricetin	$C_{15}H_9O_8^-$	317.03009	317.02919	2.828	315.0728, 288.1987, 178.0880	Flavonoid
36	18.3	Isorhamnetin	$C_{16}H_{11}O_7^-$	315.05096	315.04993	3.286	270.4666, 151.2001, 108.4734, 107.7366	Flavonoid
37	18.7	9,12,13-Trihydroxy-10,15-octadecadienoic acid	$C_{18}H_{31}O_5^-$	327.21762	327.21660	3.134	211.1333, 229.1323	Fatty acid
38	19.1	Octadecanedioic acid	$C_{18}H_{33}O_4^-$	313.23865	313.23734	4.187	312.9743, 291.3503, 270.8432, 215.0090	Fatty acid
39	19.3	Hydroxyoctadecatrienoic acid	$C_{18}H_{29}O_3^-$	293.21228	293.21112	3.953	274.5020, 221.1547, 183.1214, 171.0813	Fatty acid

UHPLC-MS equipment for *L. radicans* analysis in negative ESI QTOF mode

The separation and identification of the compounds present in the extracts were performed on a UHPLC-ESI-QTOF-MS system equipped with UHPLC Ultimate 3000 RS with Chromeleon 6.8 software (Dionex GmbH, Idstein, Germany) and Bruker maXis ESI-QTOF-MS with the software Data Analysis 4.0 (all Bruker Daltonik GmbH, Bremen, Germany). For the analysis, the samples were probed to be the most efficient dilution for the detection of all metabolites. So, 100 µL of the conventional and non-conventional extracts were diluted in 400 µL of methanol, and 3 µL was injected into the equipment. The chromatographic equipment consisted of a quaternary pump, an autosampler, a thermostatted column compartment and a photodiode array detector. Elution was performed with a binary gradient system with eluent (A) 0.1% formic acid in the water and eluent (B) 0.1% formic acid in the acetonitrile. The gradient was programmed as follows: Solvent A: 88%,

decreased to 1% at 15 min, followed by 3 min of isocratic elution with 1% of solvent A and increased to 88% at 18.2 min (total elution time 20 min). Separation was carried out with a Thermo 5 m C18 80 Å column (150 mm x 4.6 mm) at a flow rate of 0.3 mL/min. ESI-QTOF-MS experiments were recorded in negative and positive ion mode, and the scan range was between 100 and 1200 m/z. Electrospray ionization (ESI) conditions included a capillary temperature of 200 C, a capillary voltage of 2.0 kV, a dry gas flow rate of 8 L/min, and a nebulizer pressure of 2 bar. The experiments were performed in automatic MS/MS mode. The structural characterization of secondary metabolites was based on HR full MS, fragmentation patterns, and comparisons with the literature data.

Determination of ORAC

75 mM PBS Buffer pH 7.4 For one liter of buffer, weigh 3.97 g of NaH2PO4 and 6.532 g of Na2HPO4 and make up to 1000 ml with deionized H2O. 18 mM AAPH Prepare 5 minutes before use.
For 5 ml of PBS buffer, add 25 mg of AAPH. 108 nM Fluorescein Prepare a 1 mM fluorescein stock solution. To do this, weigh 1.88 mg in 5 ml of PBS buffer. Then take 2.16 µl of the stock solution and make up to 20 ml with PBS buffer for a final concentration of 108 nM.

Microplate reaction:

Add-45 µl of sample or standard, Add-175 µl of 108 nM fluorescein, Incubate at 37°C for 30 min.

Add-50 µl of 18 mM AAPH, Read for 2 h every 2 min at an excitation wavelength of 480 nm and an emission wavelength of 520 nm.
The curve ranges from 1-20 µM in microtubes.

Controls:

Blank: (No fluorescein)
-Control: No inhibitor
-Control: Trolox

Determination of Ferric Reduction Complex (FRAP)

Reagents:
-12 mM sodium acetate trihydrate buffer, pH 3.6: Mix 1.64 g of CH3COONa with 16 mL of glacial CH3COOH for 1 liter of solution (make up to volume with distilled water).

-10 mM TPTZ solution (2,4,6-tripyridyl-s-triazine): Weigh 0.031 g into a 10 mL flask and make up to the mark with diluted HCl (40 mM). It is important to prepare this solution on the same day it will be used.
-20 mM $FeCl_3·6H_2O$ solution: Weigh 0.054 g of FeCl3·6H2O into a 10 mL flask and make up to the mark with distilled water.

-FRAP reagent: Mix 1020 µL of pH 3.6 buffer, 100 µL of 10 mM TPTZ, and 100 µL of 20 mM $FeCl_3.6H_2O$.

Microplate reaction:

Add 10 µL of sample (Standard), Add 290 µL of FRAP reagent, Wait 60 min, then read at 593 nm.

Determination of Antioxidant Capacity Using DPPH radical bleaching

Reagents:

Prepare 156 µM DPPH stock solution (methanol/ethanol)
Prepare the sample in methanol/ethanol (dilutions as needed)
Prepare a gallic acid curve from 20-200 µM in microcentrifuge tubes

Microplate Reaction
Add-50 µl (standard or sample), Add-150 µl DPPH Incubate for 30 min in the dark, then measure the absorbance at 517 nm

Determination of Antioxidant Activity Using ABTS radical bleaching
Reagents:
Prepare ABTS stock solution by mixing ABTS and sodium persulfate in distilled H2O, considering a final concentration of 7 mM ABTS and a final concentration of 3.6 mM sodium persulfate (Na2S2O8). Let the reaction incubate at room temperature and in the dark for 24 h.
Then, the following day, dilute the ABTS stock solution to a final concentration of 169 µM.
Prepare the sample in methanol/ethanol (Dilutions as needed)
Prepare a gallic acid curve and quercetin curve from 20-200 µM in microcentrifuge tubes

Microplate Reaction
Add-50 µl (standard or sample), Add-150 µl of ABTS
Incubate for 30 min in the dark, Then measure the absorbance at 732 nm

α-Amylase inhibition Protocol
Reagents:
Phosphate buffer (20 mM pH 6.9)
For 250 ml deionized water
-456.1 mg NaH_2PO_4*H_2O
-240 mg Na_2HPO_4
-87.7 mg NaCl
DNS 96 mM NaK 40%

First, a 0.5 N NaOH solution is weighed.
Then, 40 g of NaK is added little by little to 80 ml of this NaOH, followed by 2.19 g of DNS and then diluted to 100 ml (dissolved with constant stirring and temperature) until the solution is completely homogeneous.
Starch substrate 0.5%
-250 mg of starch soluble in deionized water
-Dissolve carefully in a water bath, Add-0.5 mg/ml α-amylase enzyme, Add-2.5 mg in 5 ml of buffer
Reaction (perform in triplicate):
Add 100 µl of sample, Add 100 µl of enzyme, incubate for 10 min at 25°C, Add 100 µl of starch
Incubate for 10 min at 25°C, Add 200 µl of DNS, Place samples in a water bath (90°C) for 5 min
Cool to room temperature, then read at 540 nm

α-Glucosidase inhibition Protocol
Reagents:

100 mM phosphate buffer pH 6.9, In 200 ml of deionized H_2O
Add -2.30 g NaH_2PO_4
Add-1.19 g Na_2HPO_4
0.5 U/ml α-glucosidase
Make a stock solution of 10 U/ml (1 mg/ml)
-Add 250 µl and dilute to 5 ml of buffer
Add 5 mM P-nitrophenyl-α-D-glucopyranoside
-Weigh 0.0075 g in 5 ml of deionized H_2O
200 mM Sodium Carbonate (Na_2CO_3)
-Mass 1.06 g of Na_2CO_3 in 50 ml of deionized H_2O
Reaction in test tubes:
Add-600 µl of 100 mM phosphate buffer pH 6.9, add -250 µl of p-NPG, add -100 µl of sample

Preincubate 5 min at 37°C, add -50 µl of 0.5 U/ml α-glucosidase enzyme

Incubate 15 min at 37°C, add -1000 µl of Na_2CO_3, Read at 400 nm

AChE and BuChE Enzyme Inhibition Analysis

Reagents: 50 mM Tris-HCl buffer, pH 8.0, For 500 ml
Add-Trizma base 3.0238 g , add -37% HCl 1.1 ml

3 mM DTNB
For 100 ml (in buffer)
Add-0.1138 g of DTNB
Add-0.585 g of NaCl
Add-0.466 g of $MgCl_2$

5 mM AChE substrate
Add-7.23 mg in 5 ml of deionized water
Add 0.3 U/ml AChE enzyme
For 5 ml of buffer
Add -3 ul of stock (500 U/ml) *

*0.28 U/ml BuChE enzyme
For 5 ml of buffer
Add-13 ul of stock (100 U/ml)
Add 5 mM BuChE substrate
Add -7.93 mg in 5 ml of deionized water *

*NOTE
Blank (add everything except the enzyme)
Control (-) (Add everything except the sample)

Reaction:

On a kinetic plate, Add-25 ul sample, add -125 ul DTNB, add -25 ul Enzyme

Incubate for 15 min at 37°C, add -25 ul substrate, Read for 20 min every 1 min at 412 nm and 37°C

Determination of Total Phenolic Content

Reagent Preparation
- 10% (v/v) F-C Reagent: Mix 1 mL of RF-C in 10 mL of distilled water.
- 5% (w/v) Na_2CO_3: Mix 5 g of Na2CO3 in a 100 mL flask and make up to the mark with distilled water.

Microplate reaction:
- 10 µL sample (standard)
- 150 µL H_2O
- 12.5 µL Folin's reagent

Incubate for 5 min at 37°C, add - 37.5 µL 5% $NaCO_3$, Incubate for 30 min at 37°C, then Read at 765 nm

Curve: Gallic acid (GA) as standard (20–160 µg/mL methanolic solutions)
